# Co‐designing resources to support older people with intellectual disabilities and their families plan for parental death and transitions in care

**DOI:** 10.1111/hex.14000

**Published:** 2024-03-03

**Authors:** Rebecca Anderson‐Kittow, Richard Keagan‐Bull, Jo Giles, Irene Tuffrey‐Wijne

**Affiliations:** ^1^ Faculty of Health, Science, Social Care and Education, School of Nursing Kingston University Kingston upon Thames UK

**Keywords:** ageing, co‐design, intellectual disability, parent carers, planning

## Abstract

**Background:**

Older people with intellectual disabilities and their families report a lack of support for planning for parental death and transitions in care. This article aims to demonstrate the process of co‐designing resources to support older people with intellectual disabilities and their families to plan for the future.

**Methods:**

Following interviews and focus groups with older people with intellectual disabilities and their families, we used an adapted experience‐based co‐design process to develop planning ahead resources. This included a ‘trigger film’ summarising findings from the earlier interview study, 12 co‐design workshops and a user feedback phase.

**Results:**

The co‐design group developed a set of 102 ‘Planning Ahead Cards’ to help families to talk about the future and prepare for meetings with social care professionals. The group made decisions about the content, format and design of resources, and how co‐design workshops would run. The user feedback phase led to changes to the cards, and families and stakeholder groups suggested that they would be useful for planning ahead.

**Conclusion:**

The Planning Ahead Cards may facilitate planning for parental death and transitions in care for older people with intellectual disabilities and their families. The co‐design approach was key to ensuring that the resources were useful and accessible for families.

**Patient or Public Contribution:**

People with intellectual disabilities and their families contributed to the design of the resources through the co‐design workshops and feedback phase. The research team includes a research assistant with intellectual disabilities who co‐facilitated co‐design workshops and co‐authored this article.

## 1 BACKGROUND

The importance of planning for transitions in the lives of people with intellectual disabilities (known in the United Kingdom as ‘learning disabilities’) has repeatedly been highlighted in research literature and government reports.^1^, ^2^ However, the focus is often on transitions from childhood to adulthood, with later life emphasised less.^3^ As life expectancy for people with intellectual disabilities increases,^4^ more parents care for their adult children at home into older age. This can lead to the person with intellectual disabilities and their elderly parents living together, each with their own complex care needs. Without plans in place, families can hit crisis points and people with intellectual disabilities can lose both their parent and their home following parental death.^5^, ^6^ Regardless of potential crises, people with intellectual disabilities should have the support to live independent lives just as their nondisabled peers do. There is a lack of targeted resources for this group of families, many of whom have never engaged with social services and find themselves navigating this system for the first time.^7^, ^8^

There is growing support for the principle that resource development should be driven by people who will use those resources. This has led to increased recognition of the importance of co‐production methods in research and service development,^9^ resulting in a range of new approaches. Experience‐based co‐design (EBCD)^10^ aims to make meaningful changes to services by centring service users' experiences and insights. It involves making a film representing stakeholder views, using this as a starting point to identify priorities and working together to co‐design changes to services. EBCD and similar approaches have been used in a range of settings, including emergency medicine, cancer services, mental health services and primary care.^9^, ^11^

The co‐production collective identifies being ‘challenging (and disruptive)’ and ‘working towards social justice’ as key values for co‐produced research, highlighting its potential to require academic ‘experts' to step aside and give a voice to others.^12^, ^13^ For this reason, co‐design may be a particularly valuable approach when designing resources for people with intellectual disabilities and older carers, as these groups have historically been excluded from decision‐making processes, both in research and in society more generally. Involvement in co‐design work can also have benefits beyond the project itself, including increased skills, knowledge and confidence.^14^

The terms ‘co‐design’ and ‘co‐production’ are increasingly being used in research, but studies reporting these methods differ in the level of involvement of the co‐producers involved.^12^, ^14^ Involvement can sometimes be shallow and tokenistic; time and reporting pressures of research projects can mean that processes lack the flexibility required to enable genuine co‐design.^15^ Detailed descriptions of the process of co‐designing resources together with people with intellectual disabilities and their families, including decision‐making processes that led to the final resources, will be valuable for future researchers.

### 1.1 Previous phases of the project

This study was part of a wider project (*‘*Growing Older, Planning Ahead’) aimed at improving support for older people with intellectual disabilities and their families.^1^ PPI groups including people with intellectual disabilities and family carers were involved in developing the project proposal.

In an earlier project phase, interviews and focus groups were conducted with 36 people with intellectual disabilities, parents and siblings about their experiences, hopes and concerns about future planning. Participants were recruited through ‘gatekeepers’ at family carer and support organisations in South East England, and calls on social media. Due to the requirement to be able to provide informed consent, all participants with intellectual disabilities would be described as having mild to moderate intellectual disabilities, although their support needs varied. In contrast, family members had relatives with a wider range of intellectual disabilities, including those with severe and profound disabilities.

Analysis showed that families knew that they needed to plan ahead, but they did not feel supported to do so. They were concerned that their relative needed to be valued as a person, maintaining control and independence. They found that there was a lack of suitable future options and feared that there were no viable alternatives to living with family, with a view that social services only step in at crisis point (reference withheld).

## 2 AIMS AND OBJECTIVES

This article aims to demonstrate the process of co‐designing planning resources with people with intellectual disabilities and families.

The aim of this study was to co‐design resources to support older people with intellectual disabilities and their families to plan for parental loss and transitions in care. There were three objectives:

1. Identify key priorities for resources from the perspectives of people with intellectual disabilities and family carers.
2. Explore the best format for these resources to ensure that they are useful and acceptable for people with intellectual disabilities and their families.
3. Gain feedback about the usefulness of the resources for planning.

## 3 METHODS

### 3.1 Design

The design is an adaptation of EBCD, as developed by the Point of Care Foundation,^10^ including a trigger film, co‐design workshops and a feedback phase (see Box 1).

BOX 1. **Adapted EBCD stages**

1. *Trigger film*: Creating a film of people with intellectual disabilities and family carers discussing their experiences, hopes and concerns about future planning.
2. *Co‐design workshops*: Group sessions to co‐design resources to aid planning for older adults with intellectual disabilities and family carers.
3. *Feedback phase*: Introducing resources to families and stakeholder groups to obtain feedback on usefulness, accessibility and impacts on planning for the future.

### 3.2 Sample and recruitment

The inclusion criteria were as follows:

*People with intellectual disabilities*: Over 35 years old; living at home with parent(s) and able to provide informed consent.

*Family carers*: Parent, sibling or other close relative of a person with an intellectual disability who is over 35 years old and living at home with parent(s).

In the earlier project phase, the age limit was 40 years, not only because the life span of people with intellectual disabilities is shorter than the general population but also because people in their 40s are likely to live with ageing parents. However, findings suggested that the issues around future planning arose earlier, when people with intellectual disabilities stayed in the parental home beyond early adulthood. For this reason, the age limit was lowered to 35 years.

Participants for the co‐design group were purposively selected from among people who took part in the earlier interview/focus group study, and who were asked if they were willing to be contacted with further study updates and information. Those who consented were sent information about the co‐design part of the study in video and written formats, including easy‐read. Participants were selected to include families at different stages of the planning process and parents of people with mild, moderate and profound intellectual disabilities. Eleven people consented to participate in the co‐design group: Five people with intellectual disabilities, five parents and one sibling. Eight were able to regularly attend meetings. Two sets of participants were from the same family (with both the person with an intellectual disability and their parent taking part).

The resources were aimed primarily at family carers, but if their family member with an intellectual disability was able (and wanted) to give informed consent, they were included as study participants in the feedback stage. For this stage, participants took part in two different ways: (1) Those who took part in the earlier study, but not the co‐design group, were invited to test the resources within their family; calls for further participants were made on social media and through relevant carer groups and day service providers. Seven parents, one sibling and four people with intellectual disabilities were recruited. Participants were financially compensated for their involvement using their choice of vouchers or bank transfers. (2) Relevant established groups of stakeholders known to the research team were approached and asked to look at the resources with people with intellectual disabilities and provide feedback. These groups were the PPI panel for (*wider project—removed for review*); a group of older people with intellectual disabilities funded to find out about their peers' lives; and 3 day services with older service users and/or family groups interested in future planning.

### 3.3 Research team

The research team included R. A.‐K., an experienced qualitative researcher, responsible for project management; R. K.‐B., a research assistant with intellectual disabilities; J. G., a research assistant supporting R. K.‐B. and I. T.‐W., principal investigator. We reflect on how our roles and experience impacted the co‐design process in Section 4.4.

### 3.4 Procedure

#### 3.4.1 Trigger film

The 20‐min trigger film was structured around issues arising in interviews and focus groups:

1. When should we plan?
2. What are the options?
3. Can I keep my independence?
4. Who will help us to make plans?
5. Will it work?
6. Who will support us in the future?

Five participants from the interview study were filmed speaking about these topics. This was used to trigger discussions within the co‐design workshops.

#### 3.4.2 Co‐design workshops

Workshops were planned iteratively during the co‐design process to allow flexibility to focus on what group members thought was important. Twelve workshops took place online (due to the geographical spread of participants) between December 2021 and July 2022. Two group members who could not use Zoom came into the research office to join the online workshops. Workshops were co‐facilitated by R. A.‐K. and R. K.‐B. An Easy Read Agenda was circulated before meetings and written and audio minutes were sent afterwards. Every workshop started with ice‐breaker games that the research team joined in with. Group members were sent signs to indicate if they wanted to speak, needed a break and when people used jargon. Box 2 presents the content of each workshop.

BOX 2. **Overview of workshops**

1. Introductions, working together (ground rules), clarifying aims of the group, watching the first part of the trigger film, discussion in breakout rooms.
2. Watching more of the trigger film and discussing in breakout rooms, deciding how the group will work together.
3. Format and topics for resources.
4. Content and design of resources.
5. Feedback on initial prototype resources.
6. Guest speakers 1 and 2: Shared Lives service user and carer.
7. Guest speaker 3: Social worker.
8. Guest speakers 4 and 5: Community‐based intellectual disability service manager and service user.
9. Guest speaker 6: Head of ‘life planning’ organisation.
10. Feedback and edits to the full set of prototype resources.
11. Further edits to prototype resources; initial designs from illustrator.
*Seven‐week gap between workshops, during which co‐design group provided ongoing feedback about design, illustrations and content of resources*.
12. Finalising resources.
*Celebration event following the final workshop including games, a quiz, group members bringing in their art and time to socialise. Each group member was presented with a goodie bag, including the finished resources, and a card thanking them for their contribution*.

Workshops were recorded to allow researchers to return to group discussions between sessions, ensuring that decisions made by the group were enacted. Anonymous feedback about how the group was run was collected after the third and twelfth workshops via Easy Read questionnaires.

#### 3.4.3 Feedback phase

The group developed a set of ‘Planning Ahead Cards’ and a booklet to write down outcomes of discussions (see Section 4.1). As the final part of the co‐design process, we gathered feedback from families and stakeholder groups.

*Family feedback*: Participants were sent the following by post and/or email:

1. *Baseline questionnaire* including quantitative and free‐text questions about (a) concern about the future, (b) preparedness for the future and (c) steps taken to plan for the future.
2. *Planning Ahead Cards and booklet* with written and video instructions.
3. *Feedback questionnaire* to complete after using the cards, including information about how they used each card, what they liked/did not like and thoughts, feelings or actions it prompted. Family members and people with intellectual disabilities could complete this together.
4. *Follow‐up questionnaire* to complete 2 months after receiving the cards. This contained the same questions as the baseline questionnaire and further feedback on the cards.

Families were asked to use the cards on at least one occasion over 2 months.

*Stakeholder group feedback*: Following a call with R. A.‐K., stakeholder groups were sent the resources and asked to give informal feedback over email about their usefulness and what changes were needed. The *Growing Older, Planning Ahead* PPI group also gave feedback during the development of the cards.

The feedback phase was conducted between September 2022 and February 2023.

## 4 RESULTS

### 4.1 Overview of resources

The initial proposal for this study suggested that the co‐design group would develop a decision‐aid resource. However, it became clear during the workshops that this approach was not appropriate. There is no clear series of decisions to make, as housing and support options will differ over time according to individual circumstances, local provision and funding. Support was needed a step before decision‐making. Families wanted to feel equipped to articulate what they wanted and needed when talking to the professionals who had the authority to enact these decisions.

The group therefore developed a set of 102 ‘Planning Ahead Cards’ to help families to think and talk about what is important to them now and in the future. The cards, alongside a booklet to write down key points from discussions, were designed to help families to prepare for meetings with social care professionals to discuss future plans.

Cards are A5 and include a picture, label and prompts for discussions. There are seven categories of cards:

1. Things I like to do.
2. Things I might need help with.
3. Home: What is important?
4. People in my life.
5. About me.
6. Talking about death and dying.
7. Information cards.

They are available in physical and online formats. Ideas for how the cards and booklet could be used are provided in written format with the physical cards and in a video on the online version.

The following sections outline how these cards were developed over 12 co‐design workshops and following the feedback phase. Additional quotes are provided in Appendix 1 to further demonstrate decision‐making by the group.

### 4.2 Decision‐making during workshops

#### 4.2.1 Group format

In workshops 1–2, the group split into breakout rooms: one with people with intellectual disabilities and one with family members. These groups discussed their reactions to the trigger film and then came back together to feedback about their discussions. We were unsure about whether group members would feel comfortable sharing their views within a mixed group (e.g., would someone with an intellectual disability be happy to share the negatives of living with their parents in front of other parents?). However, at the end of the second workshop, the group unanimously chose to remain working as one group, feeling that it was important to hear from each other and work together:

Without the other group telling us what they feel and what they want, we'd perhaps discuss away from what they're thinking. They're the ones it's all about. (Fern, Mother)

I'm happy with a bigger group. (Sharon, Person with an intellectual disability)

While workshops were online, the group chose to hold a celebration event in person so they could meet and celebrate their achievements together.

#### 4.2.2 Inviting guest speakers

The EBCD process usually includes professionals as part of the co‐design group.^10^ However, interviews showed that many participants had little contact with professionals and that they wanted help to prepare before having conversations with social care professionals. We therefore invited relevant professionals as guest speakers, rather than group members. Speakers were invited based on what the group thought would be helpful (see Box 2 for details of six guest speakers):

Someone to tell us about the different options of the places you could live in. (Dane, Person with an intellectual disability)

Someone who knows about your rights of what you are entitled to when you're moving ahead. (Fern, Mother)

Brief talks by guest speakers were followed by questions from group members. The purpose of the guest speaker sessions was not to promote specific services or service models, but rather, to help the group to understand key issues that may need to be included in the resources and ideas about how families can advocate for themselves in assessments or discussions with social workers. Group members fed back that these guest speakers were helpful in the development of the resources, but also for their own personal thinking and planning:

Excellent—so valuable—a real perk of being part of this group. (Postsession 12 feedback questionnaire)

#### 4.2.3 Format/approach of resources

Feedback from the trigger film showed that families thought that it was difficult to plan due to uncertainty about their future support needs and difficulties working with social care professionals. In their experience, people are made to fit around what funding and support is available, rather than support fitting around their needs. They wanted resources to help them navigate this process, prepare for meetings with social workers and make their case for what support is needed:

How can we plan ahead, what can we put in place to feel prepared and say, this is what we want before the assessment? Could there be some template or something to support this, saying this is who I am, this is what I want? (Sofia, Sister)

We therefore discussed different possible formats for resources to address this need, including information/case study films, booklets, decision‐aids and conversation starters. The group felt that information booklets can become out of date as things quickly change. However, they agreed that the best approaches to planning (i.e., starting with what is important to the person) do not change. We presented an existing set of conversation cards^16^ designed to help support workers talk with people they support and ensure that they provide the support they want and need. The group liked this approach and decided to make a set of cards to help families think and talk about the important things to tell social workers, along with a template booklet to record discussions:

I think you need to be a little bit clear yourself before you engage with a social worker and that's why I think these cards are a very good example of how to start conversations… My daughter would find it quite difficult to think about just straight off what she would want… I think something that would help get the conversation started. (Alison, Mother)

Yeah [the cards] would be helpful. (Andy, Person with an intellectual disability)

#### 4.2.4 Design of the cards

The group wanted accessible resources that would help to start conversations. They therefore agreed that the cards should include pictures, labels and prompts for things to talk about. An initial set of prototype cards was developed using a widely used photo library featuring actors with intellectual disabilities. While the group thought that these images represented the topics well, they preferred commissioned illustrations that were viewed as more attractive and allowed the group to have input into images as they developed:

I think [photos] give the impression you're talking about those particular people whereas the drawing can direct their focus where you want it to go… Your sketches would get the conversation going. (Anthony, Father)

In the 7‐week break between workshops 11 and 12, the group was sent images over email and WhatsApp so that they could suggest changes to the design and illustrations. Three group members with intellectual disabilities came in person to view the proposed drawings. Changes were made to hairstyles and clothes to make people look less ‘dowdy’, more people with mobility aids, more people from different ethnic backgrounds and changes to individual pictures that were unclear. Box 3 shows examples of comments from group members that informed the development of the cards.

BOX 3. **Comments about the design of resources**

I think having tips on the back of what to talk about might be great as well because it's easy to forget about something important and get side‐tracked. (Alison, Mother)

I think it would be best to have both [pictures and headings] because some people can understand the picture and some people can understand the words. (Sharon, Person with an intellectual disability)

I need the big letters. (John, Person with an intellectual disability)

People are going to feel tense doing it, but something that makes it feel a bit cosy and nice to approach, that's what the drawings do. (Alison, Mother)

He looks nervous. She looks more like a carer than a friend. She's leaning over him. (Rochelle, Person with an intellectual disability)

It's a bit one‐sided. (Dane, Person with an intellectual disability, discussing the ethnicity of characters in illustrations)

Get rid of any abbreviations like e.g. (Fern, Mother)

#### 4.2.5 Topics covered by cards

Group members highlighted the need for the cards to cover things you want to do, not just daily support needs. Similarly, they agreed that the cards should prompt you to think about the little things that are important but you might forget:

My daughter really likes to get the newspaper. That's the sort of thing we'd probably forget to tell people but it makes a big difference to her week… It would probably come up in the cards and you'd think oh yeah we need to jot that down. (Alison, Mother)

The group identified key issues to include in the conversation cards, including:

1. What are all the big and small things that are important in your life?
2. What are all the big and small things you need help with?
3. What would happen if you didn't get this help?
4. What types of places to live would be appropriate or not and why?

We brought mocked‐up examples of topic cards for the group to try out. This helped us to see the types of conversations that the cards elicited and what prompts might be helpful. They also made suggestions for further topics:

How to pay a bill. (Sharon, Person with an intellectual disability)

Lock on my door… My girlfriend come to stay. (John, Person with an intellectual disability)

In the next round of revisions to the cards, there were a lot of cards that could be overwhelming, but the group did not think that anything should be removed:

It seems like a lot but you've got a lot to cover. It's good to have choice and pick out the relevant ones. (Fern, Mother)

Instead, they suggested clear written and video instructions you do not need to look at all of the cards and can use them in different ways.

The final decision was to call the cards ‘Planning Ahead Cards’ and the booklet ‘Me and my plans’.

### 4.3 Feedback phase

Feedback from families and stakeholder groups is presented below.

#### 4.3.1 Content

Feedback suggested that users felt that the topics covered were comprehensive. Not all cards were relevant to all families and the number of cards could seem overwhelming at first, but they fed back that they covered what was needed:

Everyone's situation is different and the cards covered a very broad area, so people will be able to pick and choose what they need. (Sasha, Mother)

The detailed prompts helped families to think about aspects of planning that are not always addressed:

Questions have been well thought out and they will help to make people think! The ‘what would happen if’ and ‘things you don't like to do’ questions are excellent because often planning ahead tends to only focus on what the person likes and hardly ever analyses the impact of not getting the right help for the person. (Sasha, Mother)

The ‘Talking about death and dying’ set was the most challenging for several participants. Families struggled to talk about this and sometimes had to switch to a different topic, but they felt that it was important to include it:

Martin got a bit upset about losing his dad, but he's got to face the other part about losing me. (Vera, Mother)

#### 4.3.2 Design and accessibility

The design of the cards and booklets was well received by family participants and stakeholder groups. Feedback about the bright colours and range of drawings was particularly positive. Including pictures of people can be difficult as it is hard to ensure that the person looking at them feels that they represent them and the people in their lives, but feedback suggested that a range of different people were represented:

Good that there are people in wheelchairs and black and white people. And all the pictures of the different religions. You've got the old person and then the young person. You've got a person on a walker and a person not on a walker. I like that because there's lots of different people with disabilities. (Vera, Mother)

Her son Martin added:

I liked the girlfriend and boyfriend one—it's like me and my girlfriend.

However, there was some negative feedback about certain cards, particularly in the ‘People’ section:

Picture not good‐old lady. (Jill, Mother, referring to ‘Mum’ card)

Views on accessibility of the cards differed. Feedback suggested that the size of the cards and the pictures increased accessibility and made the cards enjoyable. However, the prompts on the back were criticised for not being accessible to all people with intellectual disabilities. The prompts were designed to be used by family carers to help guide their conversations with their relative with an intellectual disability. However, stakeholder feedback suggested that people with intellectual disabilities may want to use the cards on their own and that this would require a simpler format:

Some people would like to look at the cards on their own so that they can think about them and make their own decisions… Some people may be able to read the words but not understand what they mean. (Stakeholder feedback, Older people with intellectual disabilities group)

Similarly, some family participants said that it would be easier to involve their son/daughter when using the cards if prompts were more accessible.

#### 4.3.3 Impact on planning

Descriptive data on family participants' self‐reported planning and concerns about the future at baseline and follow‐up are presented in Table 1.

**Table 1:** Descriptive baseline and follow‐up data on future planning.

	Baseline	Follow‐up
	Mean (range)	Mean (range)
Level of concern about family's future living/caring situation^a^	6.8 (1–10)	6.3 (1–10)
Level of preparedness for future changes in family's circumstances (including living and caring set‐up)^a^	5.5 (3–10)	5.5 (2–10)
Extent to which family has taken steps or action for planning ahead^a^	5.9 (2–10)	5.3 (3–9)
Extent to which planning steps/actions have been influenced by the use of the planning cards^a^	N/A	8.0 (6–10)
Helpfulness of cards^a^	N/A	8.3 (6–10)

Abbreviation: N/A, not applicable.

^a^ On scale of 1–10.

These quantitative data show little change in average scores on these items, but free‐text responses provided a more nuanced understanding. At baseline, several participants rated their steps towards planning ahead highly, but free‐text responses revealed that these steps had not led to progress with future plans:

I have tried to contact residential units but have got no response. I do not know where to look! We just go round in circles and I then give up. I have made significant steps but nothing has happened. (Sarah, Mother)

While some participants' scores improved, others showed increased concern and/or decreased preparedness after using the cards because they had prompted them to think about plans that they needed to make. Gill, a mother whose concern score increased from 1 to 8 but preparedness increased from 4 to 6, stated:

Talking to my daughter has made me realise we need to sit down and have a proper family chat.

Free‐text feedback suggested that the cards have the potential to aid future planning. This included both short‐ and longer‐term planning. For instance, the ‘Things I like to do’ cards led to discussions about short‐term plans like going on holiday or starting to attend a day centre. Others added more detail to existing plans:

I'd never thought about writing about how my daughter's moods are and what help she would need during her period but the questions on the cards acted as prompts and really helped me to include more detailed information in her “Hospital Passport.” (Sasha, Mother)

They helped families to think more clearly about what is needed to plan for the future:

It helped me to think about my role in the future… Clearer about what Tom needs to stay at home. Realistic about what that would look like. A bit more positive. (Sarah, Mother)

The cards have certainly brought to the fore how much there is to take into account when life decisions have to be made for future care. (Stephanie, Sister)

They also brought up discussions that led to people with intellectual disabilities expressing their wishes for the future:

I would want to live with [sister] in the future. (Fred, Person with an intellectual disability)

Some participants commented that while the cards helped with their thinking, planning still seemed daunting due to the lack of support to turn plans into real changes:

Professional support is very limited… Professionals need to be involved as parents need to be able to explore all options in depth and detail. (Sarah, Mother)

#### 4.3.4 Actions following feedback

The key negative feedback was the lack of accessibility of the prompts on the back of the cards. Discussions in the co‐design group and feedback about the benefits of existing prompts meant that we did not want to reduce level of detail. Instead, we developed new cards with two to four ‘Quick Questions’ for each topic set that could be used when families did not want to use the detailed prompts. Information about using the cards was updated to explain how to use the prompts.

The cards were designed to be used by families in advance of meeting with professionals to feel prepared for these meetings. However, feedback showed that some families felt limited in what they could do alone to plan for the future. The information has therefore been updated to include reference to involving professionals using the cards and to highlight ‘Ideas and Tips’ and ‘Information’ cards that give suggestions for putting plans into action and finding the right support. The cards have also been included in online courses for both families and professionals.

Other suggestions about the content of individual cards have been implemented (e.g., adding a question about the type of person you would want to live with to the ‘Living with other people’ card).

### 4.4 Reflections on researcher roles within the co‐design group

R. A.‐K. planned and co‐facilitated workshops and was present in all interviews in the first phase of the study, meaning that she had the most knowledge and control about the content of co‐design workshops. This potential power imbalance was addressed by sharing the interview study findings through the trigger film and flexibility to base workshop content around what group members thought was useful. The feedback questionnaire helped to address these issues. One person stated ‘I am not as clear on aims and deadlines as those of who you leading the project’, leading to further discussion of plans for the group in the next workshop.

R. K.‐B., who has an intellectual disability, played a key role in co‐facilitating the group. He describes his reflections on his role: ‘I think that me giving my own experiences helped people to be able to talk a bit more. Some people were surprised I was living as well as I was living. Sometimes it was quite difficult because you had to keep it quite professional and you were wondering how much information you should give someone. It was like making a cake. The group thought of the ingredients they would like to put in it and me, Becky [R. A.‐K.], Irene [I. T.‐W] and Jo [J. G.] cooked it! We gave it to the group to try and test them to see if they liked what came out of the oven! We had to adapt it and change some of the recipe’.

J. G.'s main role was to support RK‐B during the workshops. This was vital to ensure that he had the right support in place to co‐facilitate the group. I. T.‐W. designed the original study and had a supervisory role during the co‐design process, providing feedback and expertise as the cards developed, particularly when addressing illness and dying.

## 5 DISCUSSION

In this paper, we have described the process of co‐designing resources to support future planning for older people with intellectual disabilities and their families. These resources were based on evidence from interviews and focus groups with people with intellectual disabilities and family members; 12 co‐design workshops; a user feedback phase; and PPI input throughout this and the wider project. This approach follows the principles of shared decision‐making, inclusivity and person‐centred care that are vital in intellectual disability research and practice.^17^, ^18^, ^19^ We have transparently documented our processes to evidence how decisions were made. Such transparency is important to evidence an inclusive approach as opposed to token representation, and to allow others to learn from our experiences.^15^, ^20^

Co‐design workshops showed that families were frustrated with the barriers to future planning and wanted to feel prepared for meetings with social care professionals. This fits with previous research that showed that families were aware of the need to make plans but did not engage in planning due to a lack of support and viable options.^3^, ^21^, ^22^ This resulted in the development of a set of ‘Planning Ahead Cards’ designed to help families talk about their wants and needs for the future. Conversation starter card and workbook approaches to planning have been received positively in other contexts, such as for advance care planning.^23^, ^24^, ^25^ Visual approaches to starting conversations and decision‐making with people with intellectual disabilities, such as Talking Mats, have also been shown to be effective.^26^, ^27^

Feedback suggests that people with intellectual disabilities and their families found the Planning Ahead Cards helpful and that they have the potential to impact future planning. They helped to focus discussions and direct families to think in detail about what is needed to plan for their future. While experiences of families of people with intellectual disabilities vary considerably, families felt that the cards captured their experiences and needs.

### 5.1 Facilitators to the co‐design process

There were several factors that helped the group run smoothly and ensure that the resources were genuinely co‐designed. One challenge for co‐design projects can be balancing meaningful participation with time and resource constraints.^28^ Having 12 workshops over 8 months meant that there was time to listen to group members and make changes based on their feedback. This was a longer timeframe than originally planned (6 months) and meant adjusting plans for the feedback phase, but ensuring maximum input into resource development was prioritised. Similarly, having the flexibility to move away from the anticipated decision‐aid approach meant that the group was not constrained in what they could create. During the feedback phase, participants rarely used the feedback forms exactly as intended (e.g., giving feedback in an email rather than completing the form). Rather than chasing up the exact format that we had requested, we allowed this flexibility as it resulted in rich data that focused on what research participants, not the research team, thought was important. These sorts of deviations from planned protocols are not always comfortable for researchers but can result in more useful data and reduce burdens on participants. Flexible approaches to gathering feedback are recommended and have been used in other co‐design projects.^15^, ^29^

Other helpful elements included allowing time for the group to get to know each other. This was particularly important in the online setting, as people did not have opportunities for incidental conversations as they arrived or left. All group members had taken part in interviews so had met some of the research team, and ice‐breaker games helped group members get to know each other. The celebration event and comments on the feedback questionnaire showed the value of in‐person meetings. In future co‐design projects, arranging at least one in‐person event during the design process may facilitate group relationships.

Group members were particularly positive about workshops involving guest speakers. Reciprocity is a key principle of co‐production^30^ and this was a tangible benefit of being part of the group. It may have benefited the process to ask the group before the workshops if they wanted to invite any professionals to be group members. It is usual in EBCD to include professional input and this can help to bring different groups together and build trust outside of the health and social care environment.^10^, ^20^

### 5.2 Strengths, limitations and future research

A key strength of this study is that the co‐design method put people with intellectual disabilities and their families at the centre of the process of designing resources. This included people with limited experience with services or research, avoiding the problem of contributions being limited to ‘super‐users' who regularly take part in research studies.^15^, ^31^ Understanding the needs of people who have not been engaged with services previously is particularly important when designing resources for people who have lived at home into older age. A further strength of this paper is that the co‐design process is reported in detail, including the voice of co‐design group members through quotes from group meetings.

An effort was made to include diverse experiences within the group in relation to the levels of disability and stages of planning. However, a small group can never represent all situations, and there is a risk of bias towards the experiences of those present. There was, for example, a lack of ethnic diversity within the group, with most participants from white British backgrounds.

The flexible approach taken during co‐design meetings is a strength of our study. However, this meant more constraints during the feedback phase. We asked families to use the cards over 2 months, but this created artificial timelines for families. They are designed to be used over months and years and so the limited time available within the research project made obtaining real‐world world feedback difficult. Asking families to complete lengthy questionnaires was also an additional burden to people whose time was already stretched. An alternative approach would be to obtain initial PPI feedback, followed by an option for users to provide ongoing feedback as they use the cards (not asking for time consuming questionnaires to be completed). This would require partnering with an organisation who can continue updating the resources beyond the end of the research project, an approach that would also aid better dissemination.

The Planning Ahead Cards were designed to be used within families in the United Kingdom, but feedback suggests that they could be used by professionals such as support workers, day centre workers and social workers. They could also be used beyond the United Kingdom as the issues raised have been reported globally.^32^ Future research could explore these possibilities and evaluate their use in different contexts. A longitudinal evaluation would more clearly indicate the cards' impact on future planning.

### 5.3 Conclusion

This article documents the process of co‐designing resources to support future planning for older people with intellectual disabilities and their families. The clear documentation of decision‐making processes provides evidence for the inclusive approach and ideas for others using co‐design methods with people with intellectual disabilities. Feedback shows that the co‐design process worked well and that the Planning Ahead Cards are useful and acceptable. The cards and booklet have been made freely available for families and professionals.

## AUTHOR CONTRIBUTIONS

**Rebecca Anderson‐Kittow**: Conceptualisation; methodology; data curation; investigation; validation; formal analysis; project administration; writing—original draft; writing—review and editing. **Richard Keagan‐Bull**: Methodology; investigation; formal analysis; writing—review and editing. **Jo Giles**: Methodology; investigation; validation; formal analysis; writing—review and editing. **Irene Tuffrey‐Wijne**: Conceptualisation; methodology; investigation; validation; formal analysis; supervision; funding acquisition; writing—review & editing.

## CONFLICT OF INTEREST STATEMENT

The authors declare no conflict of interest.

## ETHICS STATEMENT

Co‐design workshops received ethical approval from London—Camberwell St Giles Research Ethics Committee (REC reference 21/LO/0494). Family testing in the feedback phase received ethical approval from Yorkshire & The Humber—Bradford Leeds Research Ethics Committee (REC reference 22/YH/0138). Pseudonyms are used throughout this paper and other potentially identifying information has been removed or changed. All participants provided written informed consent.

## Supporting information

Supporting information.

## Data Availability

There is limited data that can be shared without risking confidentiality. However, additional data that support the findings in this study are available in the supplementary information.
