# Supplementary material for: Co‐designing resources to support older people with intellectual disabilities and their families plan for parental death and transitions in care
Source: Health Expect. 2024 Mar 3;27(2):e14000. doi: 10.1111/hex.14000 (PMC10909614; doi:10.1111/hex.14000)
Supplement: Supplementary file 1 — Supporting information. [file HEX-27-e14000-s001.docx]

# **Appendix 1. Quotes demonstrating decision-making processes in the co-design group**

The tables below include quotes from co-design group members during workshops and from feedback questionnaires completed after the third and twelfth workshops.

Table 1. Decisions about group format

| **Decision** | **Quotes** |
| --- | --- |
| Do not split into separate groups for people with intellectual disabilities and family members. | It’s nice to have the opinions of both groups at the same time and hear from each other. (Linda, Mother)  Without the other group telling us what they feel and what they want, we’d perhaps discuss away from what they’re thinking. They’re the ones it’s all about. (Fern, Mother)  I’m happy with a bigger group (Sharon, Person with an intellectual disability)  You ain’t alone in a group there are others who listen and will tell their stories (Post session 3 feedback questionnaire)  I thought this was well managed by the team- those that had not spoken were invited to give their views. I always felt I was able to say what I wanted (Post session 12 feedback questionnaire)  [Working in smaller groups within meetings] worked well - Would have liked more opportunities to do this- so clever how zoom could be used to send you into a group (Post session 12 feedback questionnaire) |
| Hold workshops on Zoom but the celebration event in person. | I feel [the workshops] work really well. Especially as due to Covid it has all been done on Zoom which is sometimes hard for PwLD (Post session 3 feedback questionnaire)  First time using zoom for this type of work and think it was harder as did not have the informal chats that happen before and after face-to-face meetings- and maybe travelling together/grabbing a coffee outside of meeting (Post session 12 feedback questionnaire)  We’d be keen to come in and meet everyone in person [for the celebration event] (Linda, Mother) |

Table 2. Decisions about guest speakers to invite

| **Decision** | **Quotes** |
| --- | --- |
| Inviting guest speakers generally | Very good, essential for the project (Post session 12 feedback questionnaire)  Excellent- so valuable- a real perk of being part of this group (Post session 12 feedback questionnaire) |
| Inviting intellectual disability service providers and users | Someone who maybe does provide this sort of thing. Someone on the other end really because they’ll have a view on it which would be interesting… they’ll know more about the limits as well (Anthony, Father)  Someone to tell us about the different options of the places you could live in (Dane, Person with intellectual disabilities) |
| Inviting a social worker | Social worker (Linda, Mother, in response to being asked who to invite)  Someone who knows about your rights of what you are entitled to when you’re moving ahead (Fern, Mother)  [The social worker’s] contribution was invaluable, the session with him was enormously helpful (Anthony, Father) |
| Inviting ‘life planning’ practitioner | Fern (Mother): When you’re person-centred planning you’re in the centre and it’s all about you. Everything’s planned around you and all the people are around you.  Andy (Person with an intellectual disability): Because I’m just worried about people stopping you  Fern: Yeah so it’s all about what you want not about what the people around you want, it’s all about you.  Andy: Oh so it’s not about what the carer wants?  Fern: No it’s about you and people should listen.  John: About Andy  R1: And there are people out there who will go through that process of planning with you. Would it be helpful to have someone who does that come to one of these meetings?  Andy: Yeah that would be good |

Table 3. Decisions about format/approach of resources

| **Decision** | **Quotes** |
| --- | --- |
| Resources should help families to plan for assessments and conversations with social workers or local authorities. | It’s getting harder and harder to work with social services, their funding is limited (Alison, Mother)  Someone said if you’re getting a social worker, be careful which one you get (Andy, Person with an intellectual disability)  How can we plan ahead, what can we put in place to feel prepared and say, this is what we want before the assessment? Could there be some template or something to support this, saying this is who I am, this is what I want? (Sofia, Sister) |
| Create a set of conversation cards to help families to think and talk about plans for the future. Include a template to write down results of these discussions and bring to meetings with social workers. | Think of all the different questions that would make them think what they want. Like John doesn’t like noise from other people. Then you could write in that book and then if you had your meeting with your social worker, you can say, this is what I want. (Fern, Mother)  I think you need to be a little bit clear yourself before you engage with a social worker and that’s why I think these cards are a very good example of how to start some conversations... My daughter would find it quite difficult to think about just straight off what she would want… I think something that would help get the conversation started really. (Alison, Mother)  Yeah [the cards] would be helpful (Andy, Person with an intellectual disability)  Something to write notes in (John, Person with an intellectual disability) |

Table 4. Decisions about design of resources

| **Decision** | **Quotes** |
| --- | --- |
| Include pictures, headings and conversation prompts (using large text). | I think the pictures and the words and then the words can be your heading for focusing your discussion points for when you’re going to meet a social worker… I think having tips on the back of what to talk about might be great as well because it’s easy to forget about something important and get side-tracked (Alison, Mother)  I think it would be best to have both [pictures and headings] because some people can understand the picture and some people can understand the words (Sharon, Person with an intellectual disability)  I need the big letters. (John, Person with an intellectual disability) |
| Have picture and text in separate white boxes. | I like it in the box (John, Person with an intellectual disability)  The box one seems clearer though, that’s like the picture there and the subject underneath” (Andy, Person with an intellectual disability) |
| Use illustrations not photos. | I think [photos] tend to give the impression you’re talking about those particular people whereas the drawing can kind of direct their focus where you want it to go... Your sketches would get the conversation going (Anthony, Father)  People are going to feel tense doing it, but something that makes it feel a bit cosy and nice to approach, that’s what the drawings do (Alison, Mother)  Photosymbols is well known with people around the country… but it’s not exhaustive. When you want something a bit different it’s harder (Fern, Mother) |
| Changes to initial illustrations. | Love the black and white one, colour ones are ok but guess would cost more and not sure they’re better (Alison, Mother).  He looks nervous. She looks more like a carer than a friend. She’s leaning over him (Rochelle, Person with an intellectual disability, looking at initial ‘meeting new people’ illustration)  It’s a bit one-sided (Dane, Person with an intellectual disability, discussing the ethnicity of characters in illustrations) |
| Changes to make cards easier to use | Get rid of any abbreviations like e.g. (Fern, Mother)  I think the layout was really good. The ideas and tips are good but I think maybe they should be separate. I could get waylaid into looking stuff up rather than actually concentrating, (Alison, Mother)  It would be better all on one side, it’s distracting turn it over all the time (Anthony, Father)  In my head I’d be doing it online. I assume it would be easier to fill in online… it’s easier to change it and to send it to other people (Alison, Mother) |

Table 5. Decisions about topics covered in resources

| **Decision** | **Quotes** |
| --- | --- |
| Detailed prompts to elicit what is important to you, what help you need, and what would happen if you don’t get the right support.  Sacrificing brevity to allow prompts that encourage detailed thinking over range of topics. | Little things like my daughter really likes to get the newspaper. That’s the sort of thing we’d probably forget to tell people but it makes a big difference to her week… It would probably come up in the cards and you’d think oh yeah we need to jot that down. (Alison, Mother)  If they were playing away from home then I’d need help (Andy, Person with an intellectual disability, trying out the ‘going the events’ card and talking about help he would need to go and watch sport)  It seems like a lot but you’ve got a lot to cover. It’s good to have choice and pick out the relevant ones (Fern, Mother)  There is a lot on the back of the cards but I think it’s needed (Alison, Mother) |
| Topics and prompts both about what is important in life now and also changes for the future. | I’m just wondering if this gets people too focused on what they’ve already got and wanting to repeat it. Whether you need to start opening the idea that perhaps you could have something different in the future. (Alison, Mother)  We’re talking about plans for the future so obviously experiences of where you live now would be crucial, but the main objective is what happens next… I think ‘where I live now’ is too specific, maybe ‘what’s important’. (Anthony, Father) |
| Include information cards not just conversation starters. | What about financial advice? You know what it costs and how much money you can have in the bank before you get the help (Linda, Mother)  Information about what your rights are about choosing where you want to live. (Fern, Mother) |
| Additional topics and prompts added. | How to pay a bill (Sharon, Person with an intellectual disability)  Lock on my door… My girlfriend come to stay (John, Person with an intellectual disability)  Something about sex and relationships… it’s always the thing people forget about (Fern, Mother)  Periods or how you might cope with them, or the dentist and healthy diets (Alison, Mother) |
